# Neonatal Intestinal Obstruction: Etiology, Management, and Outcomes in a Tertiary Care Center

**DOI:** 10.7759/cureus.62971

**Published:** 2024-06-23

**Authors:** Shrikesh Singh, Saurabh Srivastav, Neeti Agarwal, Amit Nagpure, Tanvir R Khan

**Affiliations:** 1 Paediatric Surgery, Dr Ram Manohar Lohia (RML) Institute of Medical Sciences, Lucknow, IND; 2 Obstetrics and Gynaecology, Mayo Institute of Medical Sciences, Lucknow, IND; 3 Pediatric Surgery, Sawai Man Singh (SMS) Medical College, Jaipur, IND

**Keywords:** etiology, neonate, mortality, post-operative complications, intestinal obstruction

## Abstract

Background: Intestinal obstruction in neonates remains a critical medical emergency in the field of pediatric surgery. Clinical conditions often experience a sudden deterioration in their appearance. Multiple factors contribute to unfavorable clinical outcomes in underdeveloped nations. The study was conducted to identify the etiology, management, and outcomes of neonatal intestinal obstruction at a specialized medical facility.

Methods: This retrospective study included 168 newborns who had to be operated on in the neonatal intensive care unit between 2021 and 2023 due to intestinal obstruction. The clinical and demographic characteristics of the infants, final diagnosis, surgical complications, and mortality rate were documented. In addition, the relationship between risk factors such as birth weight, gestational age, length of surgery, and postoperative problems was evaluated.

Results: The majority of neonatal intestinal obstruction occurred within seven days of birth, with 8-15 days being the second most common. Most babies were born at full term (53.57%) and weighed 2 kg or more (75%). In newborns in our region, duodenal, ileal, jejunal, and colonic atresias were found to be the most common causes of neonatal intestinal obstruction that requires surgery. The study detected 45 postoperative problems, 26.79% of the total. Out of 168 patients, twelve (7.14%) had septicemia, seven (4.17%) had anastomotic leak, seven (4.17%) had aspiration pneumonitis, and two (1.19%) needed re-exploration. Overall mortality was 10.12%, with 17 patients dying. Moreover, 119 patients (70.83%) survived without issues, while 32 (19.05%) survived with complications.

Conclusion: Our findings emphasize the significance of promptly diagnosing, intervening, and implementing suitable management approaches to enhance outcomes for newborns with intestinal obstruction. Furthermore, it highlights valuable perspectives for healthcare professionals in enhancing care for this specific group of patients.

## Introduction

Intestinal obstruction in newborns occurs in approximately 1 out of every 2000 live births, making it a common cause for admission to neonatal intensive care units [1,2]. Hirschsprung's disease, malrotation, volvulus, atresia, and other conditions like necrotizing enterocolitis (NEC) are the primary causes of intestinal obstruction in neonates. In addition, meconium ileus and meconium plug syndrome are commonly observed as reasons [3,4]. The primary indications of intestinal obstruction in a neonate typically include bilious vomiting, distended abdomen, absence of meconium within 24 to 48 hours, and signs of intestinal obstruction such as diminished or absent bowel sounds. Newborns may also display symptoms of shock or dehydration, excessive crying, and difficulty with feeding [5,6].

The presumed diagnosis is based on the presence of abdominal distension, bilious vomiting, absence of meconium discharge within the first day of life, and the occurrence of polyhydramnios throughout pregnancy [6,7]. The attending physician can determine the correct diagnosis by doing a comprehensive medical history and physical examination and confirming it with simple radiological tests. A routine abdominal radiograph is often sufficient to diagnose the condition, as the gas pattern is readily apparent and often provides information about the location of the obstruction. Early diagnosis and treatment lead to improved outcomes. Neonates with intestinal obstruction may experience complications such as aspiration of vomit, sepsis, midgut infarction, or the development of enterocolitis [8].

Early treatment of intestinal obstruction patients significantly reduces morbidity and mortality rates. The presence of congenital comorbidities and the occurrence of delays in diagnosis and treatment have an impact on the final result [9].

The elevated mortality rate in less developed countries can be ascribed to various causes, such as poverty, insufficient obstetric care, inadequate equipment, delayed admission, and subpar transportation networks. Due to these factors, it is extremely difficult to effectively treat babies with intestinal obstruction in these countries [10-13]. It also provides a stimulus for further studies to explore these associations.

Newborns with intestinal obstruction need prompt diagnosis and treatment. Despite several investigations to ascertain the cause and treatment of this illness, further study is needed to identify the impacts of different management strategies. To address this knowledge deficit, a comprehensive examination of the causes, treatments, and outcomes of newborn intestinal obstruction is carried out in a specialized healthcare facility. In this study, we aim to investigate the etiology, management, and outcomes of neonatal intestinal obstruction at a specialized medical facility.

## Materials and methods

Study design

In a retrospective study, 168 newborns who were diagnosed with intestinal obstruction between 2021 and 2023 in the neonatal intensive care unit (NICU) of the Tertiary Teaching Institute of Northern India who required surgical intervention were examined. The study aimed to investigate cases of bowel obstruction in neonates requiring surgical intervention. The neonatal intensive care unit (NICU) is a specialized facility with 10 beds. It is a level III referral unit, which means that it provides advanced medical care.

Ethical considerations

This retrospective study on neonatal intestinal obstruction utilizes de-identified data from medical records maintained by the tertiary care center’s Neonatal Intensive Care Unit (NICU). The data is fully anonymized, ensuring that no personally identifiable information (PII) is accessible to the researchers. The study involves no interventions or changes in patient care, thus posing minimal or no risk to participants. The primary goal of this study is to evaluate historical treatment outcomes to inform future clinical practice and improve patient care quality within the institution. The institutional research office has reviewed and exempted this protocol, confirming compliance with all relevant ethical standards and guidelines.

Study criteria procedure

Neonates at a tertiary care center NICU were included if they had an intestinal obstruction within 28 days after delivery. Neonates who had insufficient medical records and neonates who had life-threatening conditions other than intestinal obstruction were not included in the study.

Assessments

Data on the patient's demographic and clinical characteristics, final diagnosis, surgical management and complications, and mortality rates were collected and documented in the files. The study also investigated the relationship between postoperative problems and various risk factors such as birth weight, gestational age, and timing of surgery.

Sample size calculations

Since all data and information were collected for the two years of the study, the sample size was determined using the census method, and all available records were thoroughly examined. The study included all newborns diagnosed with intestinal obstruction by a neonatologist, pediatrician, or pediatric surgeon who subsequently underwent surgery. The study excluded neonates who were initially diagnosed with bowel obstruction like hypothyroidism, sepsis with ileus, or early necrotizing enterocolitis but did not require surgery during follow-up visits. It also secluded the neonates with syndromic presentations. The patients with anorectal malformations were also excluded owing to the obvious clinical findings.

Statistical analysis

Data analysis was performed using IBM Corp. Released 2012. IBM SPSS Statistics for Windows, Version 21.0. Armonk, NY: IBM Corp. The variables analyzed were recorded using descriptive statistics, including frequency and percentage. Categorical data were analyzed using a chi-square test or Fisher's exact test, depending on the circumstances. A p-value of less than 0.05 was considered statistically significant.

## Results

The majority of cases of neonatal intestinal obstruction were observed within the first seven days after birth, with the second most prevalent age range being between 8 and 15 days. The male-to-female ratio was 91:77. The majority of patients had a birth weight of 2 kg or more 126 (75%) and were born at full term 90 (53.57%). The majority of patients, 89 (52.98%), underwent surgery within two days, while a smaller proportion, 48 (28.57%), had their surgery between 2 and 4 days. Due to a lack of awareness, antenatal scans were not done or not available in almost all of the cases.

The prevalence of abdominal distension, vomiting, excessive crying, refusal to feed, blood in stool, absence of bowel movement, and visible bowel loops at clinical presentation was in 137 (81.55%), 133 (79.17), 57 (33.93%), 78 (46.43%), 18 (10.71%), 122 (72.62%), and 32 (19.05%), respectively (Table 1).

**Table 1:** Details of baseline characteristics of the patients

Characteristic	Details	n (%)
Age of presentation	0-7 days	104 (61.90%)
	8-15 days	64 (38.10%)
	Total	168 (100.0%)
Birth weight	Weight < 2 kg	42 (25.00%)
	Weight≥2 kg	126 (75.00%)
Pre-Term/Term	Pre-Term	78 (46.43%)
	Term	90 (53.57%)
Time from admission to surgery	Within 2 days	89 (52.98%)
	2 to 4 days	48 (28.57%)
	More than 4 days	31 (18.45%)
Clinical presentation	Abdominal distension	137 (81.55%)
	Vomiting	133 (79.17%)
	Excessive crying	57 (33.93%)
	Refusal to feed	78 (46.43%)
	Blood in stools	18 (10.71%)
	Failure to pass stools	122 (72.62%)
	Visible bowel loops	32 (19.05%)

**Figure 1:**
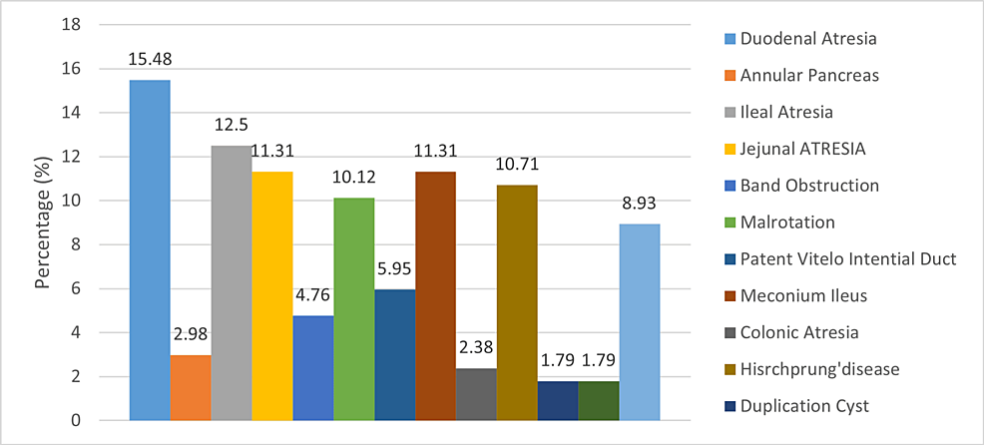
A bar chart shows the frequencies of different causes of neonatal intestinal obstruction

Of the 168 patients, a total of 26 (15.48%) had duodenal atresia, and five (2.98%) had annular pancreas. Of the total number of patients, 21 (12.50%) had ileal atresia, 19 (11.31%) had jejunal atresia, eight (4.76%) had band obstruction, and 17 (10.12%) had malrotation. Of the total number of patients, 10 (5.95%) had patent vitelo-intestinal duct, 19 (11.31%) had meconium ileus, four (2.38%) had colonic atresia, 18 (10.71%) had Hisrchprungs disease, three (1.79%) had duplication cyst, three (1.79%) had obstructed hernia, and 15 (8.93%) had necrotizing enterocolitis (Figure 1).

**Figure 2:**
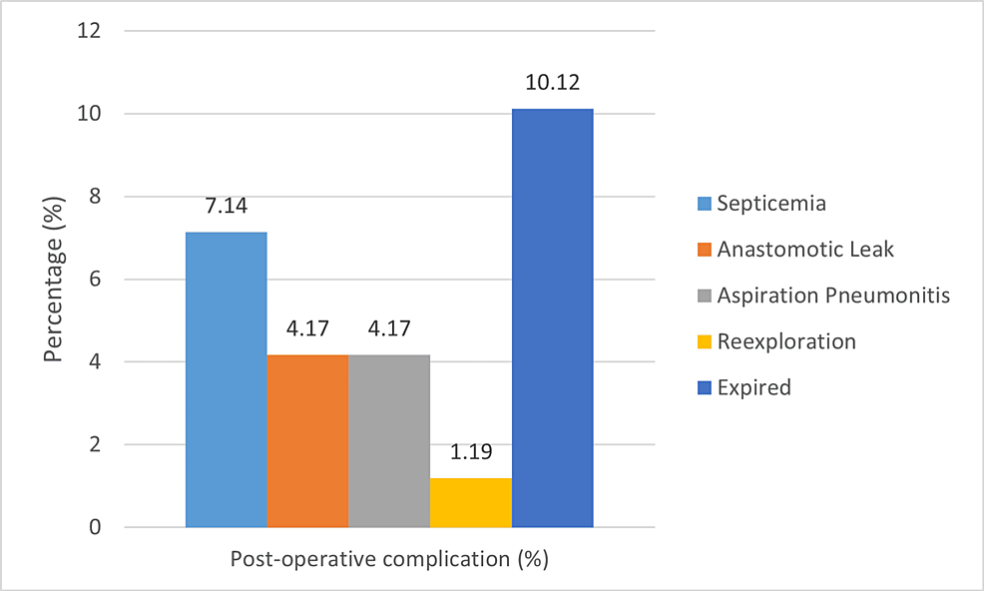
Bar chart showing the postoperative complications

The postoperative complication was found in 45 (26.79%) patients. Out of the 168 patients, 12 (7.14%) had septicemia, seven (4.17%) had an anastomotic leak, seven (4.17%) had aspiration pneumonitis, and two (1.19%) required re-exploration. Out of the 168 patients, a total of 17 individuals, accounting for 10.12% of the sample, did not survive (Figure 2).

Of the 168 patients, 119 (70.83) patients survived without complications, 32 (19.05%) patients survived with complications as mentioned above, and 17 (10.12%) patients did not survive. Table 2 shows the association of the cause of intestinal obstruction with disease-specific outcomes. The cause of intestinal obstruction was not significantly associated with disease-specific outcomes (Table 2).

**Table 2:** Association of the cause of intestinal obstruction with disease-specific outcome p-value (Chi-Square test with significant values =p<0.005 ) p= 0.87: Overall comparison p=0.956: Comparison between survived without/with complication p=0.382: Comparison between survived without complication and non-survivor p=0.745: Comparison between survived with complication and non-survivor

Cause of intestinal obstruction	Survived without complication (n=119)	Survived with complication (n=32)	Expired (n=17)
	n	n	n
Duodenal Atresia	20 (16.81%)	4 (12.50%)	2 (11.76%)
Annular Pancreas	4 (3.36%)	1 (3.13%)	0 (0.00%)
Ileal Atresia	12 (10.08%)	5 (15.63%)	4 (23.53%)
Jejunal Atresia	11 (9.24%)	3 (9.38%)	5 (29.41%)
Band Obstruction	7 (5.88%)	1 (3.13%)	0 (0.00%)
Malrotation	14 (11.76%)	2 (6.25%)	1 (5.88%)
Patent Vitelo Intential Duct	7 (5.88%)	3 (9.38%)	0 (0.00%)
Meconium Ileus	14 (11.76%)	4 (12.50%)	1 (5.88%)
Colonic Atresia	3 (2.52%)	1 (3.13%)	0 (0.00%)
Hisrchprung'disease	12 (10.08%)	4 (12.50%)	2 (11.76%)
Duplication Cyst	3 (2.52%)	0 (0.00%)	0 (0.00%)
Obstructed Hernia	3 (2.52%)	0 (0.00%)	0 (0.00%)
Necrotising Enterocolitis	9 (7.56%)	4 (12.50%)	2 (11.76%)

Table 3 shows the correlation between birth weight and outcomes specific to certain diseases. The incidence of low birth weight (< 2 kg) was significantly higher in both survivors with complications (56.25%) and non-survivors (58.82%) compared to survivors without complications (11.76%).

**Table 3:** Association of birth weight with disease specific outcome p-value (Chi-Square test with significant values=p<0.05) p=<0.001: Overall comparison p=<0.001: Comparison between survived without/with complications p=<0.001: Comparison between survived without complications and non-survivor p=0.826: Comparison between survived with complications and non-survivor

Birth weight	Survived without complication (n=119)	Survived with complication(n=32)	Expired(n=17)
	n (%)	n (%)	n (%)
Weight < 2 kg	14 (11.76%)	18 (56.25%)	10 (58.82%)
Weight≥2 kg	105 (88.24%)	14 (43.75%)	7 (41.18%)

Table 4 shows the association of pre-term/term with disease-specific outcomes. The incidence of preterm was significantly higher in both survivors with complications (65.63%) and non-survivors (70.59%) compared to survivors without complications (37.82%).

**Table 4:** Association of pre-term/term with disease-specific outcome p-value (Chi-Square test with significant values=p<0.05) p=0.002: Overall comparison p=0.009: Comparison between survived without/with complication p-0.022: Comparison between survived without complications and non-survivors p=0.974: Comparison between survived with complications and non-survivors

Age	Survived without complication (n=119)	Survived with complication(n=32)	Expired(n=17)
	n (%)	n (%)	n (%)
Pre-Term	45 (37.82%)	21 (65.63%)	12 (70.59%)
Term	74 (62.18%)	11 (34.38%)	5 (29.41%)

Table 5 shows the relationship between the time from admission to surgery and the disease-specific outcome. The time of two or more than two days from admission to surgery was significantly higher in both survivors with complications (37.50%) and non-survivors (35.29%) than in survivors without complications.

**Table 5:** Association of time from admission to surgery with disease-specific outcome p-value (Chi-Square test with significant values=p<0.05) p=<0.01: Overall comparison p=<0.01: Comparison between survived without/with complication p=0.007: Comparison between survived without complications and non-survivors p=0.946: Comparison between survived with complications and non-survivors

Time from admission to surgery	Survived without complication (n=119)	Survived with complication (n=32)	Expired(n=17)
	n (%)	n (%)	n (%)
Within 2 days	76 (63.87%)	8 (25.00%)	5 (29.41%)
2 to 4 days	30 (25.21%)	12 (37.50%)	6 (35.29%)
More than 4 days	13 (10.92%)	12 (37.50%)	6 (35.29%)

## Discussion

Timely identification and prompt surgical treatment are crucial to achieve positive outcomes in neonatal intestinal obstruction. Identification of the primary causes allows for the implementation of targeted treatment approaches tailored to the specific condition of the individual patient. Nevertheless, problems that arise after surgery remain a difficult problem, emphasizing the importance of continued monitoring and treatment to achieve the best results.

Our study found that duodenal atresia, ileal atresia, jejunal atresia, and colonic atresia are the main causes of intestinal obstruction requiring surgery in neonates in our region.

Farrokhkhani et al. (2023) found that anorectal malformation is the underlying etiology of intestinal obstruction [14]. Previous studies have consistently shown that anorectal malformations are the main factor for intestinal obstruction in Ethiopia, Uganda, India, and Bangladesh [3,15-17]. In contrast to our analysis, Baad and colleagues discovered in their imaging study that Hirschsprung's disease was the most common diagnosis in infants with intestinal obstruction [18]. Hirschsprung's disease is the second most common cause of intestinal obstruction, according to one study [14]. Meconium ileus was observed as the second most common cause of intestinal obstruction with a prevalence of 11.31%, followed by Hirschsprung's disease with a prevalence of 10.71%. Hirschsprung's disease is increasingly diagnosed in neonates in industrialized countries, whereas the reverse pattern is observed in developing countries [3,19].

Neonatal bowel obstruction shows a rapid progression. Nevertheless, rapid identification and medical intervention significantly improve outcomes [20,21]. Surgical intervention is usually required for the majority of neonatal intestinal obstructions, especially in underdeveloped countries. This is a challenge due to the advanced age (age after birth at the time of presentation) of the patients. Experienced pediatric surgeons perform surgical procedures on newborns in the neonatal intensive care unit in modern medical facilities. Several countries suffer from a shortage of pediatric surgeons and specialized facilities for neonatal intensive care. Even when pediatric surgeons are available, neonates are still at risk of anastomotic leakage, sepsis, and surgical site infection [21,22].

The study found that postoperative complications occurred in 45 patients, representing 26.79% of the total. Of the 168 patients, twelve (7.14%) had septicemia, seven (4.17%) had anastomotic leak, seven (4.17%) had aspiration pneumonitis, and two (1.19%) required re-exploration. Farrokhkhani et al. [14] demonstrated that 1.4% of infants suffered sepsis after surgery, while 0.6% had surgical site infection, 1.2% suffered hemorrhage and shock, and 1.8% suffered intestinal perforation. Due to the severity of these complications, 15 additional interventions were required.

Ogundoyin et al. [23] showed that 16 (13.7%) individuals had postoperative problems. Of these, six patients (5.1%) had gastric problems and six patients (5.1%) had wound-related complications. In addition, three patients (2.6%) developed sepsis. The length of hospital stay varied between 1 and 59 days, with a median of 14 days. The pattern of postoperative complications is consistent with trends reported in other regions of the world, with sepsis being the most common complication [25-27]. Nevertheless, the observed postoperative complication rate of 13.7% is quite low compared to rates reported by other centers [24,26-29].

In our study, the mortality rate after surgery was 10.12%, with 17 patients suffering death. In addition, 119 patients (70.83%) survived without problems, while 32 patients (19.05%) survived but suffered complications. Ogundoyin et al. (2019) showed that the mortality rate was 9.4%. The pattern of postoperative complications is consistent with trends reported in other regions, with sepsis being the predominant complication [23-26]. Nevertheless, the observed postoperative complication rate of 13.7% is quite low compared to rates reported by other centers [24,26-29]. In contrast, Farrokhkhani et al. (2023) showed that postoperative mortality was observed in seven infants (4.1%).

In our study, the incidence of patients with low birth weight, prematurity, and a time interval of two or more days between admission and surgery was found to be higher in both survivors with problems and non-survivors compared to survivors without complications. This underscores the importance of prompt identification and timely surgical treatment of neonates with bowel obstruction, particularly in low birth weight or preterm neonates. Timely identification and intervention can significantly improve outcomes and reduce the likelihood of adverse outcomes.

The limitation of the present study is that it is retrospective, subject to confounding (other risk factors may be present that were not measured), and needs further studies with a larger sample size.

## Conclusions

This study provides important insights into the causes, treatment, and outcomes of neonatal intestinal obstruction in a specialized medical facility. It found that the patients with low birth weight, prematurity, and a time interval of two or more days between admission and surgery led to survivors with complications and non-survivors, compared to survivors without complications. This underscores the importance of early identification of neonates with bowel obstruction. For better outcomes, they must receive prompt surgery, and the treatment plans should be individualized accordingly.
